# Impacts of plant root traits and microbial functional attributes on soil respiration components in the desert-oasis ecotone

**DOI:** 10.3389/fpls.2025.1511277

**Published:** 2025-02-11

**Authors:** Jinlong Wang, Guanghui Lv, Jianjun Yang, Xuemin He, Hengfang Wang, Wenjing Li

**Affiliations:** ^1^ College of Ecology and Environment, Xinjiang University, Urumqi, China; ^2^ Key Laboratory of Oasis Ecology of Education Ministry, Xinjiang University, Urumqi, China; ^3^ Xinjiang Jinghe Observation and Research Station of Temperate Desert Ecosystem, Ministry of Education, Jinghe, China

**Keywords:** autotrophic respiration, heterotrophic respiration, microbial functional attributes, plant traits, desert-oasis ecotone

## Abstract

Dividing soil respiration (Rs) into autotrophic respiration (Ra) and heterotrophic respiration (Rh) represents a pivotal step in deciphering how Rs responds to environmental perturbations. Nevertheless, in arid ecosystems beset by environmental stress, the partitioning of Rs and the underlying mechanisms through which microbial and root traits govern the distinct components remain poorly understood. This study was strategically designed to investigate Rs and its components (Ra and Rh), soil properties, and root traits within the desert-oasis ecotone (encompassing the river bank, transitional zone, and desert margin) of northwest China. Employing metagenomics, we quantitatively characterized microbial taxonomic attributes (i.e., taxonomic composition) and functional attributes (specifically, functional genes implicated in microbial carbon metabolism). Field measurements during the growing season of 2019 unveiled a pronounced decline in soil respiration rates along the environmental gradient from the river bank to the desert margin. The mean soil respiration rate was recorded as 1.82 ± 0.41 μmol m^-2^ s^-1^ at the river bank, 0.49 ± 0.15 μmol m^-2^ s^-1^ in the transitional zone, and a meager 0.45 ± 0.12 μmol m^-2^ s^-1^ in the desert margin. Concomitantly, the Ra and Rh components exhibited a similar trend throughout the study period, with Rh emerging as the dominant driver of Rs. Utilizing random forest modeling, we unearthed significant associations between microbial taxonomic and functional features and Rs components. Notably, both Ra and Rh displayed robust positive correlations with the abundance of phosphatidylinositol glycan A, a key player in microbial carbon metabolism. Partial least squares path modeling further elucidated that soil properties and microbial functions exerted direct and positive influences on both Ra and Rh, whereas taxonomic features failed to register a significant impact. When considering the combined effects of biotic and abiotic factors, microbial functional attributes emerged as the linchpin in dictating Rs composition. Collectively, these findings suggest that a trait-based approach holds great promise in more effectively revealing the response mechanisms of Rs composition to environmental changes, thereby offering novel vistas for future investigations into carbon cycling in terrestrial soils.

## 1 Introduction

In terrestrial ecosystems, the soil carbon pool represents the greatest carbon storage pool at about two to three times larger than the plant carbon pool or the atmospheric carbon pool (Chen et al., 2024; Huang et al., 2020). Soil respiration (Rs), being the primary carbon release mechanism in the terrestrial carbon cycle (Li et al., 2021), has garnered significant interest among ecologists over the past few decades, emerging as a crucial factor in global carbon cycle studies (Hashimoto et al., 2023). Rs serves as the principal conduit for transferring carbon from soil pools to the atmosphere (Huang et al., 2020), comprising two key elements: autotrophic respiration (Ra), involving CO_2_ emissions from plant roots and associated microbial activity, and heterotrophic respiration (Rh), stemming from microbial breakdown of organic matter in soil. Ra contributes between 10% and 90% to the total Rs (Yan et al., 2015). A portion of the CO_2_ captured annually via photosynthesis in terrestrial plants is immediately emitted back into the atmosphere as part of Ra, whereas the rest is temporarily sequestered in organic matter and later released through Rh (Hopkins et al., 2013). However, the complexity of the different sources of CO_2_ produced by soils and the factors influencing them makes a comprehensive understanding of Rs uncertain (Bond-Lamberty, 2018), especially in more heterogeneous arid ecosystems (Kukumägi et al., 2017; Wang et al., 2021b). Consequently, it’s essential to distinguish between the autotrophic and heterotrophic components of Rs and evaluate their responses to environmental shifts, along with their consistent contributions to Rs (Chu et al., 2023; Huang et al., 2015).

Rs variations are affected by various biotic and abiotic factors including soil microclimate (temperature and moisture), nutrients, microbes, and plant traits (Borden et al., 2021; Wang et al., 2022a, 2021b). In arid regions, soil moisture and temperature exhibit extreme variability, and the scarcity of water and nutrients makes the ecosystem highly fragile (Liu et al., 2024). For example, low rainfall and high temperatures can lead to significant fluctuations in soil water availability, which will have a major impact on soil microbial activity and root physiological processes (Furtak and Wolińska, 2023), and thus affect soil respiration (Han et al., 2024). Unlike humid regions, where soil respiration is often influenced by multiple factors such as high vegetation cover and abundant water supply (Li et al., 2024b), while the limited water resources and harsh environmental conditions make soil microclimate and nutrients the primary factors controlling soil respiration in arid areas (Ngaba et al., 2024). This difference in driving factors makes the study of soil respiration in arid areas unique and provides an opportunity to understand the specific responses of ecosystems to environmental stresses. Differences in how Rh and Ra respond to these factors may alter their roles in Rs, potentially leading to decreased water and nutrient availability (Huang et al., 2015; Zheng et al., 2021). Many studies indicate that Rh fluctuations are significantly influenced by external factors like soil microclimate, nutrient status, and soil microbes (Kang et al., 2022; Tang et al., 2020a), whereas Ra correlates more with soil’s physical and chemical properties, root morphology, and chemical makeup (Paradiso et al., 2019). Root-mediated soil organic matter decomposition and the ensuing CO_2_ release through respiration occur at the root level, involving both the plant root system and inter-root microorganisms (Adamczyk et al., 2019; Chari and Taylor, 2022). However, the microbial control over Rs components, particularly the role of inter-root microorganisms in Ra, remains largely unclear.

The vegetation in arid regions is predominantly comprised of drought-tolerant plant species, whose root structures and physiological characteristics are specifically adapted to the arid environment (Liu et al., 2024). For example, certain plants possess more developed root systems that can penetrate deeper into the soil in pursuit of water and nutrients (Li et al., 2024a). This distinctive vegetation structure exerts an impact on the sources contributing to soil respiration (Borden et al., 2021; Wang et al., 2021c), with the proportion of autotrophic respiration to heterotrophic respiration potentially differing from that in other ecosystems (Quoreshi et al., 2022). Traits or functional traits are measurable characteristics of organisms that have adapted and evolved over time in response to the external environment, affecting the organism’s survival, growth, and reproduction and thus its adaptations to and functions in the environment (Shen et al., 2019). The biogeographical distribution of traits connects an organism’s function to its habitat, enabling theoretical predictions of how organisms, communities, and ecosystems respond to environmental shifts through trait modifications (Vernham et al., 2023). Trait-based microbial ecology and plant ecology research focuses on identifying a core set of traits for microbial and plant adaptations to environmental changes to predict changes in ecosystem function (Montoya, 2023; Violle et al., 2014). Root systems, crucial for plant water and nutrient absorption, adapt to various environmental conditions, making root traits vital for sustaining diverse ecosystem processes. These traits serve as key indicators for assessing plant resilience in challenging habitats (Cortois et al., 2016; Wang et al., 2021c). Research has shown that functional traits such as root morphology, chemical composition, and physiological activity have a significant impact on microbial community structure and activity (Furey and Tilman, 2023; Rathore et al., 2023; Wan et al., 2021). Most metabolic traits of microorganisms can directly affect nutrient and elemental cycling processes in ecosystems (Martiny et al., 2015). Microbial genetic traits primarily manifest through specific functional genes or metabolic pathways present in their genomes (Escalas et al., 2019). Genomic analyses, including metagenomics, facilitate comparisons between targeted gene sequences and databases annotating gene or protein functions (e.g., KEGG), aiding in deducing the roles of these sequences in carbon cycling and microbial functionality (Dai et al., 2021). Despite methods linking root trait and microbial data (including taxonomy and metabolism) to Rs (Trivedi et al., 2013), understanding of how these subterranean traits react to environmental shifts and influence Rs components remains limited.

Desert ecosystems, covering about 22% of Earth’s land surface, significantly influence the global carbon cycle (Wang et al., 2022b). Desert-oasis ecotones, found in the extremely arid deserts of northwest China, are unique ecosystems known for their high biodiversity and primary productivity. Desert-oasis ecotones are crucial for combating wind erosion, stabilizing riverbeds, moderating local climates, preserving biodiversity, and ensuring regional ecological safety (An et al., 2023; Wang et al., 2019a, 2021a; Zhang et al., 2018c). Recently, changes in ecological processes and natural landscapes within desert-oasis ecotones, driven by regional climate shifts and escalating human activities, pose significant threats to both the ecological services of these oases and the sustainability of the regional economy (Ling et al., 2015). Desert-oasis ecotones encompass various habitats, typically transitioning from densely forested riparian zones to sparse desert vegetation (Wang et al., 2021b; Williams et al., 2006). Ecological processes shaping ecosystem structure and function can vary across these habitats (Bennett et al., 2009; Tianjiao et al., 2022). Differences in environmental habitat factors make desert-oasis ecotones ideal for assessing the response of Rs components to environmental stresses. This study employed root exclusion experiments on *Nitraria tangutorum* and *Alhagi* sp*arsifolia*, common desert plants in the Ebinur Lake basin’s desert-oasis ecotone, northwest China. The objectives were to assess (1) the proportional roles of Rh and Ra in Rs; (2) correlations between microbial taxonomy/functionality and Rs components; and (3) the impacts of soil properties, root traits, microbial diversity, and functionality on Rs components. The hypothesis posits that environmental stress decreases Rs yet enhances Rh’s relative contribution, and that Rs components exhibit greater sensitivity to microbial functional genes compared to microbial taxonomic composition.

## 2 Materials and methods

### 2.1 Study sites and experimental design

**Figure 1:**
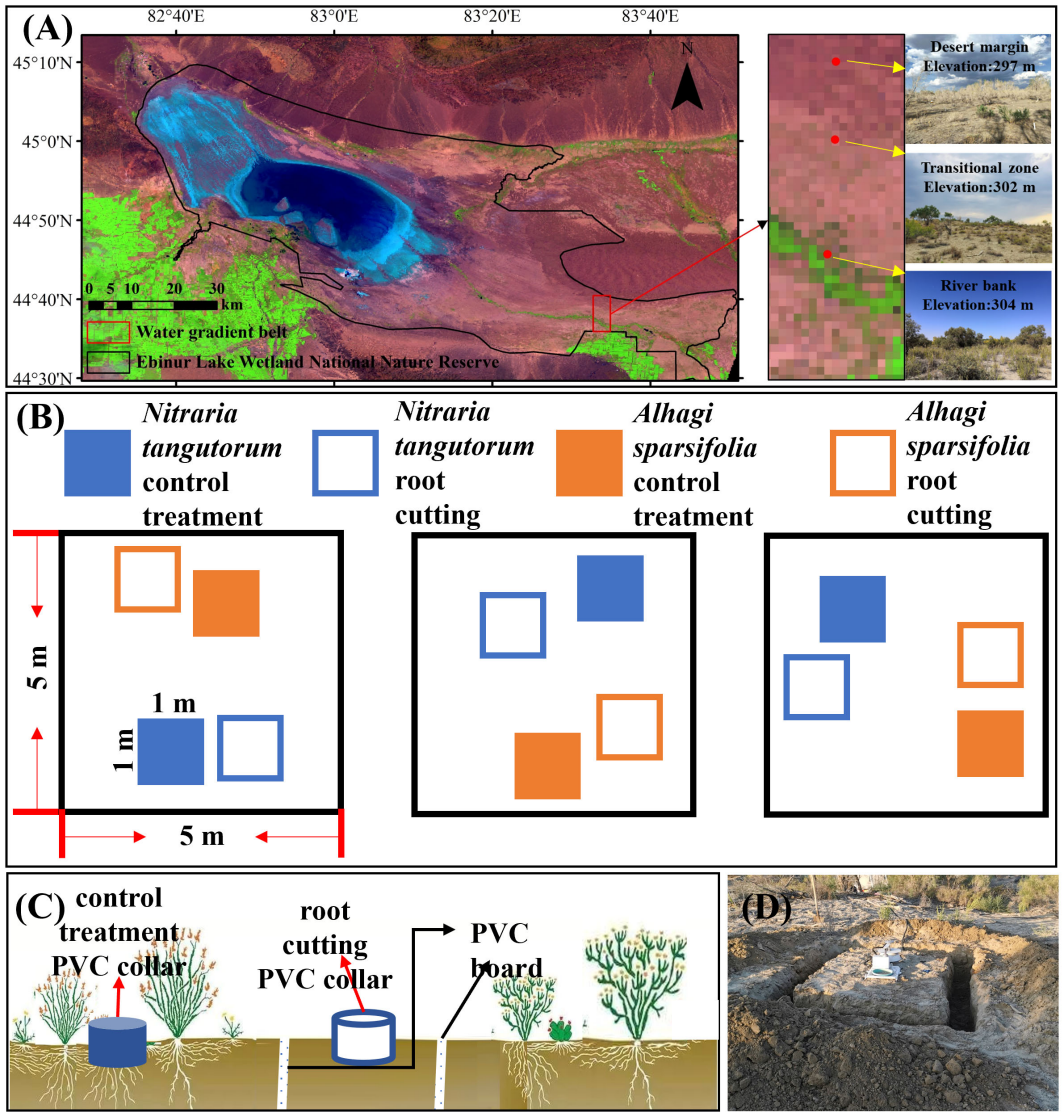
Soil respiration component partitioning experiment. **(A)** Study area and sampling point distribution; **(B)** plot layout; **(C)** schematic diagram of the root exclusion method (trenching + gap analysis) to differentiate soil respiration components. The polyvinyl chloride (PVC) collar (dark color) of the control quadrat was used to measure soil respiration, and the PVC collar (light color) of the root cutting quadrat was used to measure heterotrophic respiration. The difference between the two was autotrophic respiration. **(D)** Field *in-situ* trenching treatment.

The study site is situated in the Ebinur Lake Wetland National Nature Reserve, Xinjiang Uygur Autonomous Region, China (82°36’–83°50’E, 44°30’–45°09’N), within the Ebinur Lake basin adjacent to the southwestern border of the Gurbantunggut Desert (Fu et al., 2021). The area experiences a characteristic temperate continental arid climate, marked by minimal rainfall and significant evaporation. Dominant plant communities consist primarily of saline- and drought-tolerant species including *Populus euphratica*, *A.* sp*arsifolia*, and *N. tangutorum*. Plant distribution in the study area forms a patchy mosaic, interspersed with bare ground, offering an optimal natural setting for investigating Rs components (Zhao et al., 2011). In July 2018, a transect was set up in the Ebinur Wetland Nature Reserve, running from the Aqikesu River towards the Mutter Desert. Three habitats were identified based on their proximity to the riverbank: river bank (at a distance of 150 meters from the riverbank), transitional zone (at a distance of 3000 meters from the riverbank), and desert margin (at a distance of 5000 meters from the riverbank) (**Figure 1**) (Wang et al., 2021b).

In mid-August 2018, a randomized block experimental design method was used to set up three 5 m × 5 m blocks (replicates) in each of the three different habitats (**Figure 1**). Each habitat group included one *N. tangutorum* community and one *A.* sp*arsifolia* community as control treatments, with each plot measuring 1 m × 1 m. Additionally, a root exclusion technique (trenching combined with gap analysis) was employed to create a 1 m × 1 m root excision plot in a bare area approximately 0.5 m from the control treatment. A trench, 0.2 m wide and 0.8 m deep, was excavated around the sample (with no root presence below 0.8 m of soil), followed by the placement of a polyvinyl chloride (PVC) sheet with a 0.2 mm mesh size in the trench to prevent root intrusion into the plot. Lastly, the excavated soil was returned to the trenches (Huang et al., 2015; Lavigne et al., 2003). Ultimately, 12 plots (comprising six control treatments and six root excision plots) were set up per habitat, totaling 36 plots across all treatments within the three habitats studied.

### 2.2 Measurements of Rs and its components

A PVC collar, 20 cm in inner diameter and 15 cm tall, was centrally positioned in each quadrat, exposing approximately 5 cm above ground. The positions of these 36 collars remained unchanged during the entire measurement period. Prior to each Rs measurement, new growth was cleared from the root cutting quadrat to exclude plant respiration from the Rs readings. Within the control treatment, only above-ground live plants within the PVC collar were removed. Rs and Rh were assessed using the LI-8100 automated soil CO_2_ flux system (LI-COR, Lincoln, NE, USA) on calm, sunny days chosen mid-month from May to August 2019. All experimental measurements occurred between 10:00 and 12:00 local time on the same day, aiming to accurately reflect average daily CO_2_ emissions (Wang et al., 2021b). Each PVC collar underwent three measurements, with the average serving as the respiration rate for that location. Respiration rates in control and root excision plots were designated as Rs and Rh, respectively. Ra was determined via subtraction (Rs−Rh), with autotrophic and heterotrophic respiration ratios expressed as Ra/Rs and Rh/Rs, respectively (Borden et al., 2021). Throughout each measurement session, soil temperature (ST) was recorded at a 10 cm depth adjacent to the PVC collar, utilizing a temperature sensor (MS-10, Rain Root Technology Co., Ltd., Beijing, China).

### 2.3 Sample collection and measurement

Soil sampling occurred in mid-June 2019, with separate collections of rhizosphere and bulk soil samples. For microbial analysis, rhizosphere soil samples were taken from depths of 0–30 cm within each control treatment plot, using sterile tweezers disinfected over an alcohol lamp flame and cooled with sterile water to extract roots and remove loose soil. Once loose soil was removed, it was sealed in a sterile bag and refrigerated. Samples were transported to the lab for rhizosphere soil collection (Li et al., 2022). Root samples were moved to a 50 mL sterile centrifuge tube containing 30 mL phosphate-buffered saline (PBS) buffer (137 mmol L^−1^ NaCl, 2.7 mmol L^−1^ KCl, 8.5 mmol L^−1^ Na_2_HPO_4_, and 1.5 mmol L^−1^ KH_2_PO_4_ at pH 7.3) on a sterile operating table, where root surfaces were cleaned with sterile forceps (Edwards et al., 2015). Using sterile tweezers, the root system was extracted from the centrifuge tube, and the remaining suspension was centrifuged at −4°C (10,000 g, 1 min) to concentrate the rhizosphere soil sample. Roots extracted from the rhizosphere soil were cleaned with sterile water and dried with sterile filter paper for further root trait analysis. Surface soil was gathered from each root-cutting plot and placed in a 50 mL sterilized centrifuge tube as bulk soil. Due to insufficient DNA quantity and concentration in some bulk soil samples, metagenomic sequencing was ultimately conducted on 33 samples, comprising 18 rhizosphere and 15 bulk soils.

The soil with roots removed was split into thirds: one portion was kept in an aluminum box for soil water content (SWC) measurement via drying (105°C, 48h); another was refrigerated (4°C) for ammonium nitrogen (AN) and nitrate nitrogen (NN) content analysis; the last was air-dried for assessing soil pH, soil salinity content (SSC), soil organic carbon (SOC), available phosphorus (AP), total phosphorus (TP), and total nitrogen (TN). Total root surface area was determined with WinRhizo Pro.2013 (Reagent Instruments Inc., Canada), followed by drying roots at 65°C to a constant weight for root dry weight (RDW) calculation. Dried root samples were pulverized and sifted through a 100-mesh screen to measure plant root carbon content (RCC), root nitrogen content (RNC), and root phosphorus content (RPC). Specific root area (SRA) was computed by dividing total root surface area by RDW. Soil properties and root nutrient contents were measured according to Wang et al.’ s method (2022a).

### 2.4 DNA extraction and sequencing

About 0.2 g of rhizosphere or bulk soil was placed in a 2-mL sterile centrifuge tube, and DNA was extracted using a PowerSoil^®^ DNA extraction kit according to standard procedures. DNA integrity was assessed using a 1% agarose gel (200 V, 30 min), and DNA concentration was measured quantitatively with Qubit 2.0. DNA fragmentation was conducted with Covaris S220 based on operational parameters to create a paired-end (PE) library (Novogene Biotechnology Co., Ltd., Beijing, China). Paired-end sequencing was carried out on the Illumina Genome Analyzer (HiSeq PE150, Illumina Inc., San Diego, CA, USA). From the soil samples collected, 1,912,296,608 high-quality metagenomic DNA sequences were generated, and 17,590,216 sequences were derived through assembly and gene prediction (**Supplementary Table S2**). A total of 106 phyla, 737 families, 2,523 genera, and 16,981 species of microbial communities with definite classification information were detected. The data were submitted to the NCBI database repository under accession number PRJNA 664310.

### 2.5 Bioinformatics analysis

During data quality control, fastp software (Chen et al., 2018) was initially used to trim 3’ and 5’ adapter sequences from reads. Reads shorter than 50 bp, with an average base quality score below 20, or containing N bases were discarded, retaining only high-quality paired-end and single-end reads. For data assembly, Megahit software (Li et al., 2015), utilizing succinct de Bruijn graph principles, was used to splice and assemble optimized sequences, selecting contigs ≥ 300 bp for the final assembly outcomes. Gene prediction utilized Prodigal (Hyatt et al., 2010) to forecast open reading frames (ORFs) within contig mosaics. Genes with a nucleic acid length ≥ 100 bp were chosen and translated into amino acid sequences. All sample-predicted gene sequences were clustered with CD-HIT (Fu et al., 2012), selecting the longest gene per cluster as the representative sequence to form a non-redundant gene set. SOAPaligner (Li et al., 2008) was employed to quantify gene abundance in the relevant samples.

For taxonomic annotation of species, Diamond (Buchfink et al., 2015) compared the amino acid sequences of the non-redundant gene set against the NCBI non-redundant protein sequence (NR) database, setting the expected e-value for BLASTP comparisons at 1e-5. Taxonomic annotations for species were derived from the database matching the NR database, and species abundance was determined by summing gene abundances associated with each species. Subsequently, the abundance of carbohydrate-active enzymes was computed using the sum of gene abundances linked to these enzymes. Ultimately, 33 metagenomic data points from three locations were contrasted with the NR and carbohydrate-active enzyme databases.

### 2.6 Statistical analysis

Initially, the ‘randomForest’ package was used for random forest machine learning algorithm analysis to determine the most important microbial predictors of Rs components. The genus-level taxonomic composition and functional genes obtained using KEGG analyses based on the metagenomics of rhizosphere and bulk soil microorganisms were regressed with Rs components, and the genera or genes that contributed greatly to the prediction accuracy of the model were found by performing five rounds of 10-fold cross-validation (Zhang et al., 2018a). The ‘rfPermute’ and ‘A3’ packages enabled 1000 random permutations to assess predictor importance and overall model significance (Fortmann-Roe, 2015). Spearman correlation assessed the relationship between functional genes and Rs components. Subsequently, the ‘vegan’ package was employed for non-metric multidimensional scaling (NMDS) using Bray-Curtis distances to analyze genus-level microbial communities and functional genes, visualizing shifts in microbial taxonomy and functionality (Chen et al., 2020; Liu et al., 2019). Lastly, partial least squares path modeling (PLS-PM) was utilized to investigate the interconnections among microbial taxonomy and function (NMDS axes), root traits (SRA, RDW, RCC, RNC, RPC), soil properties (pH, SSC, SOC, TN, AN, NN, TP, AP, SWC, ST), and Rs components (Ra, Rh) via the “plspm” package (Zhou et al., 2022). A goodness of fit (GoF) index represented the overall predictive ability of the final PLS-PM model (Tenenhaus et al., 2005).

## 3 Results

### 3.1 Changes in Rs and soil properties

**Figure 2:**
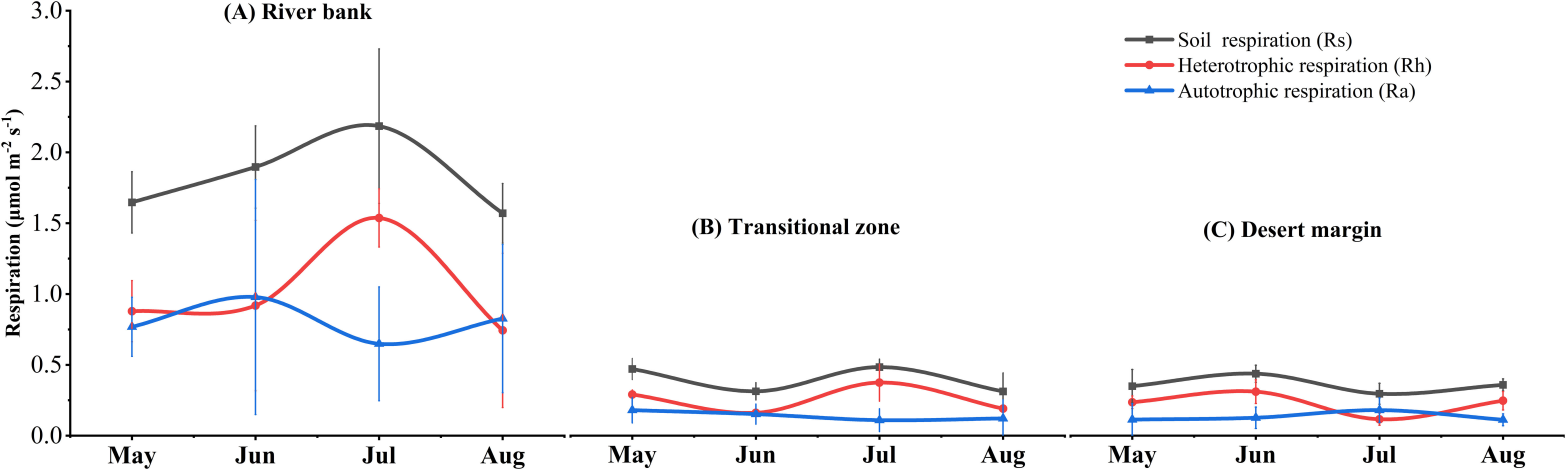
Dynamics of soil respiration and its components along the environmental gradient during the growing season (May–August). Data shown are the means ± SE of sampling plots (n = 6) from each habitat.

During the measurement process, soil respiration rates exhibited more pronounced fluctuations in the river bank habitat as compared to those in the transitional zone and the desert margin. Through one-way analysis of variance (ANOVA), it was ascertained that the rates were significantly elevated in the river bank habitat (*P <*0.05, **Figure 2**; **Table 1**). From May to August 2019, the mean value of Rs was 1.82 ± 0.41 μmol m^−2^ s^−1^ for the river bank, 0.49 ± 0.15 μmol m^−2^ s^−1^ for the transitional zone, and 0.45 ± 0.12 μmol m^−2^ s^−1^ for the desert margin (**Table 1**). Between May and August, the average contribution of Ra to Rs was 45% in river bank, 38% in the transitional zone, and 37% at the desert margin (**Figure 2C**). Compared with the river bank, the Ra in the transitional zone and the desert margin decreased by 77.8% and 79%, respectively, and the Rh decreased by 68.6% and 74.5%, respectively (**Table 1**). RCC, RNC, and RPC were the highest in the river bank plot, while SRA was the highest in the desert margin (**Table 1**). The soil in the study area had a high sand content but low silt and clay content (**Supplementary Table S1**). Sand content increased gradually from the river bank to the desert margin, while silt and clay content decreased (**Supplementary Table S1**). The environmental gradient observed primarily resulted from variations in soil properties, showing declining SWC and soil nutrients from the river bank to the desert margin as ST increased, suggesting greater environmental stress at the desert margin (**Supplementary Table S1**). Except for TN, AN, and AP, root exclusion treatment did not significantly affect other soil properties (*P* > 0.05, **Supplementary Table S1**).

**Table 1:** One-way analysis of variance (ANOVA) for soil respiration and its components and root traits along the environmental gradient.

Plot	River bank	Transitional zone	Desert margin
Soil respiration (Rs; μmol m^−2^ s^−1^)	1.82 ± 0.41 a	0.49 ± 0.15 b	0.45 ± 0.12 b
Heterotrophic respiration (Rh; μmol m^−2^ s^−1^)	1.02 ± 0.48 a	0.32 ± 0.06 b	0.28 ± 0.04 b
Autotrophic respiration (Ra; μmol m^−2^ s^−1^)	0.81 ± 0.48 a	0.18 ± 0.10 b	0.17 ± 0.07 b
Specific root area (SRA; cm^2^ g^−1^)	4.89 ± 2.22 a	2.72 ± 1.39 a	5.44 ± 4.56 a
Root dry weight (RDW; g g^−1^)	9.18 ± 4.06 b	13.86 ± 7.06 a	13.61 ± 6.16 a
Root carbon content (RCC; mg g^−1^)	246.37 ± 22.53 a	244.64 ± 15.30 a	242.48 ± 7.75 a
Root nitrogen content (RNC; mg g^−1^)	15.14 ± 3.43 a	13.31 ± 2.89 a	14.36 ± 1.74 a
Root phosphorus content (RPC; mg g^−1^)	0.85 ± 0.32 a	0.48 ± 0.09 b	0.54 ± 0.14 b

The same letters indicate no significant differences (*P* > 0.05) between different habitat types.

### 3.2 Structure and function of soil microbial communities

Soil sample bacterial abundance varied, with rhizosphere > bulk, while archaeal abundance followed the opposite pattern, rhizosphere < bulk (*P* < 0.05, **Table 2**). Across all soil samples, the dominant phyla were Proteobacteria, Actinobacteria, Bacteroidetes, Gemmatimonadetes, Planctomycetes, Chloroflexi, Firmicutes, Cyanobacteria, and Euryarchaeota. These nine dominant phyla collectively made up 79.46% of the community, with Proteobacteria and Actinobacteria being the most abundant. Proteobacteria comprised 40.77% of rhizosphere soil and 17.99% of bulk soil, whereas Actinobacteria represented 21.54% of rhizosphere soil and 40.67% of bulk soil (**Supplementary Figure S1**).

**Table 2:** Distribution of different domains obtained using MetaPhlAn analysis according to metagenomic sequence samples.

Plot	River bank		Transitional zone		Desert margin	
	Rhizosphere soil	Bulk soil	Rhizosphere soil	Bulk soil	Rhizosphere soil	Bulk soil
Bacteria	98.51 ± 0.23a	96.75 ± 1.30b	98.10 ± 1.03a	97.37 ± 0.16b	98.26 ± 0.47a	97.45 ± 0.45b
Fungi	0.11 ± 0.01a	0.09 ± 0.02a	0.14 ± 0.06a	0.11 ± 0.01a	0.11 ± 0.01a	0.10 ± 0.01a
Viruses	0.09 ± 0.06a	0.08 ± 0.02a	0.08 ± 0.04a	0.06 ± 0.01a	0.06 ± 0.01a	0.04 ± 0.01a
Archaea	1.29 ± 0.29b	3.08 ± 1.31a	1.68 ± 1.03b	2.46 ± 0.16a	1.59 ± 0.48b	2.40 ± 0.45a

The same letters indicate that there are no significant differences (P > 0.05) between rhizosphere soil and bulk soil within the same habitat type.

**Figure 3:**
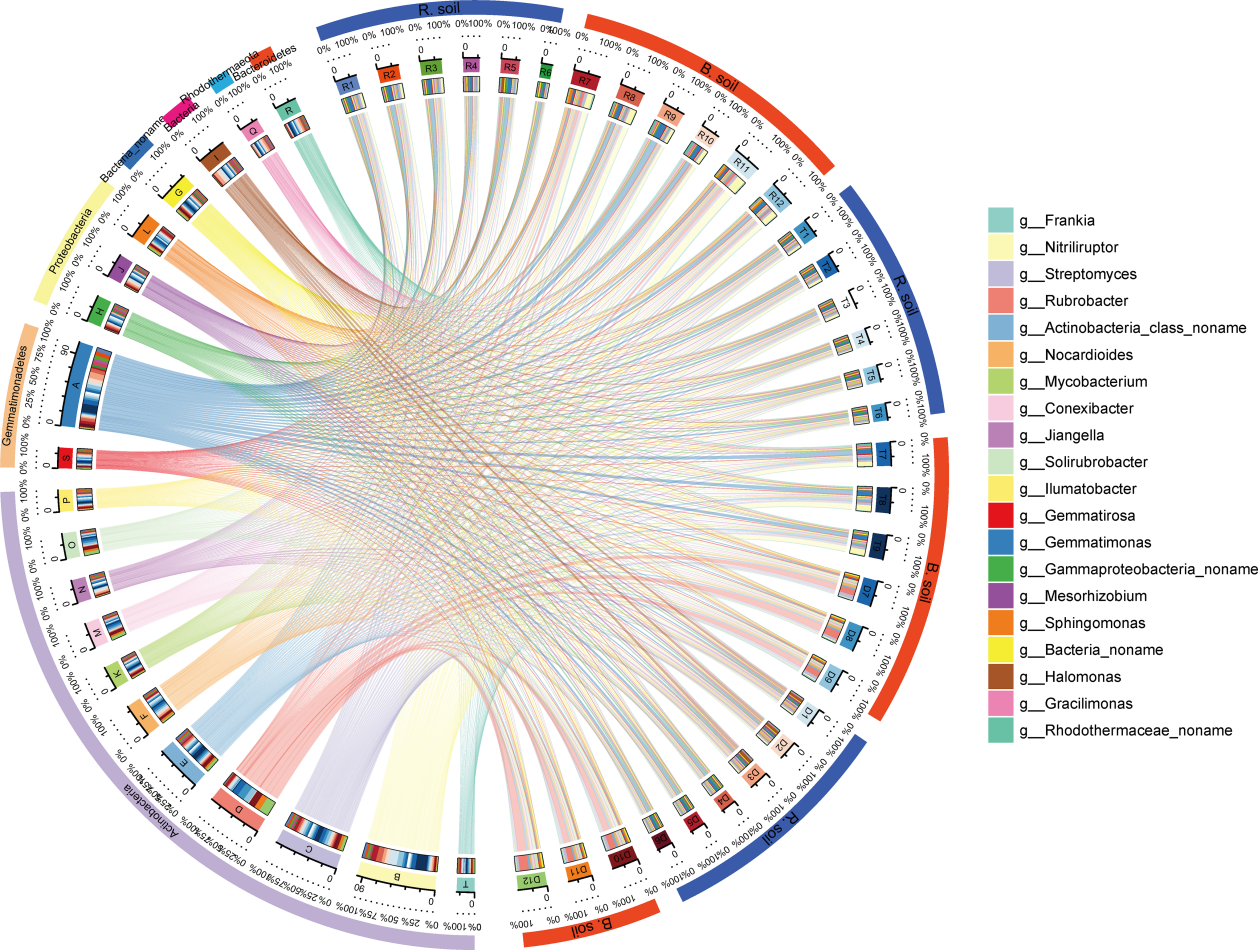
Relative abundances and proportions of dominant species in different soil samples at the genus level (showing the top 20 species with the highest relative abundances). The right semicircle represents the species abundance composition of the sample, and the left semicircle represents the distribution proportion of species in different samples. Circles from the outside to the inside depict the following: the first and second colored circles: the left half circle represents the distribution proportion of different samples in dominant species, different colors represent different samples, and the length represents the distribution proportion of the sample in a species (the percentage displayed in the second circle); the circle on the right half represents the species composition corresponding to different samples, different colors represent different species, and the length represents the abundance proportion of a species in the sample (the percentage shown in the second circle); the third circle: there is a colored band in the circle, with one end connecting the sample (right semicircle), where the end width of the band represents the abundance of species in the sample, and the other end connecting the species (left semicircle), where the end width of the band represents the distribution proportion of the sample in the corresponding species, and the value outside the circle represents the abundance value of the corresponding species. R1–R6, T1–T6, and D1–D6 are rhizosphere soil; R7–R12, T7–T9, and D7–D12 are bulk soils.

Dominant genera in rhizosphere and bulk soil varied slightly across different habitats. These dominant genera all fell within the major bacterial phyla: Actinobacteria, Proteobacteria, Gemmatimonadetes, and Bacteroidetes (**Figure 3**). Dominant genera in rhizosphere samples included *Gemmatimonas* (3.08% ± 1.10%), *Streptomyces* (1.88% ± 0.41%), *Halomonas* (1.71% ± 1.12%), *Nitriliruptor* (1.68% ± 0.86%), *Gammaproteobacteria noname* (1.59% ± 0.29%), and *Nitriliruptor* (4.43% ± 3.40%). In contrast, bulk soil samples were dominated by *Rubrobacter* (4.22% ± 3.96%), *Gemmatimonas* (3.29% ± 2.37%), *Streptomyces* (3.07% ± 0.93%), and *Actinobacteria class noname* (2.18% ± 0.62%). The relative abundances of *Gracilimonas*, *Rhodothermaceae noname*, and *Gemmatirosa* exhibited a gradual decrease from the river bank to the desert margin in both rhizosphere and bulk soil samples (**Figure 3**).

**Figure 4:**
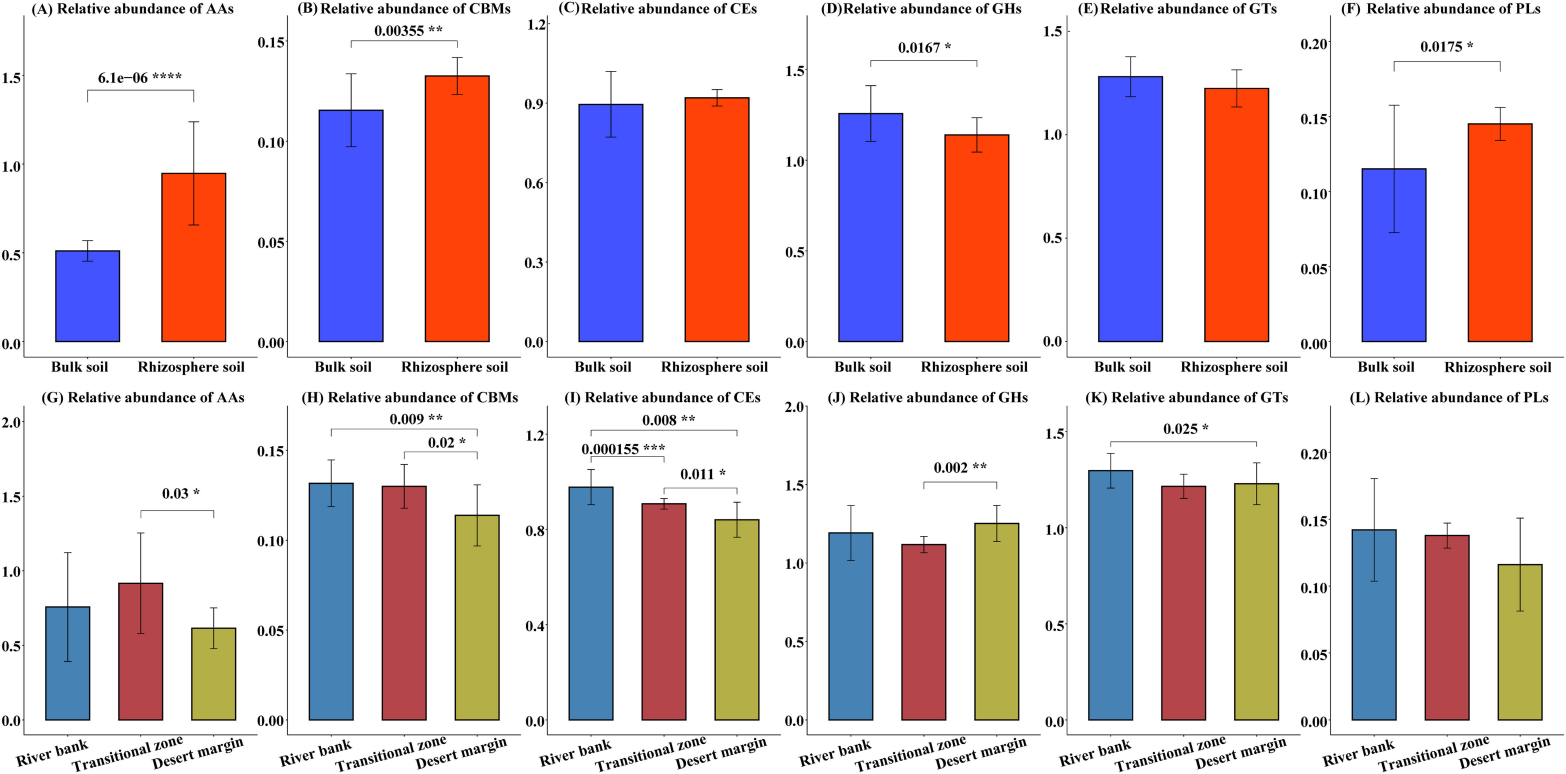
Differences in the carbohydrate enzyme gene abundance between different microhabitats **(A–F)** and plot types **(G–L)**. Glycoside hydrolases (GHs), glycosyl transferases (GTs), polysaccharide lyases (PLs), carbohydrate esterases (CEs), auxiliary activities (AAs), and carbohydrate binding modules (CBMs). GHs and GTs are involved in soil organic C decomposition and biosynthesis, respectively. PLs, CEs, and AAs are involved in the microbial decomposition of polysaccharides, carbohydrate esters, and lignin, respectively. The gene abundance of GHs, GTs, PLs, CEs, and AAs is the sum of the relative abundances of each specific gene belonging to a specific module. The numbers and asterisks above the horizontal line represent significant differences between the two groups, *P<0.05, **P<0.01, ***P<0.001, ****P<0.0001.

Metagenomic sequencing revealed a substantial presence of genes encoding carbohydrate enzymes within the soil microbial genome (**Supplementary Figure S2A**). Specifically, these genes were categorized into 109 glycoside hydrolase (GH) families, 66 glycosyl transferase (GT) families, 19 polysaccharide lyase (PL) families, 16 carbohydrate esterase (CE) families, 12 auxiliary activity (AA) families, and 41 carbohydrate binding module (CBM) families, with respective counts of 340,862; 367,148; 39,947; 259,918; 88,806 genes (**Supplementary Figure S2B**). Gene abundances for AAs, CBMs, and PLs were notably higher in rhizosphere soil compared to bulk soil, whereas GHs exhibited an inverse pattern (*P* < 0.05). Furthermore, the abundance of carbohydrate enzyme genes varied across different environmental gradients (**Figures 4G–L**). Notably, the abundance of CBM and CE genes in river bank was significantly greater than in transitional zone and desert margin (**Figures 4H–I**).

### 3.3 Identification of drivers related to Rs and its components

**Figure 5:**
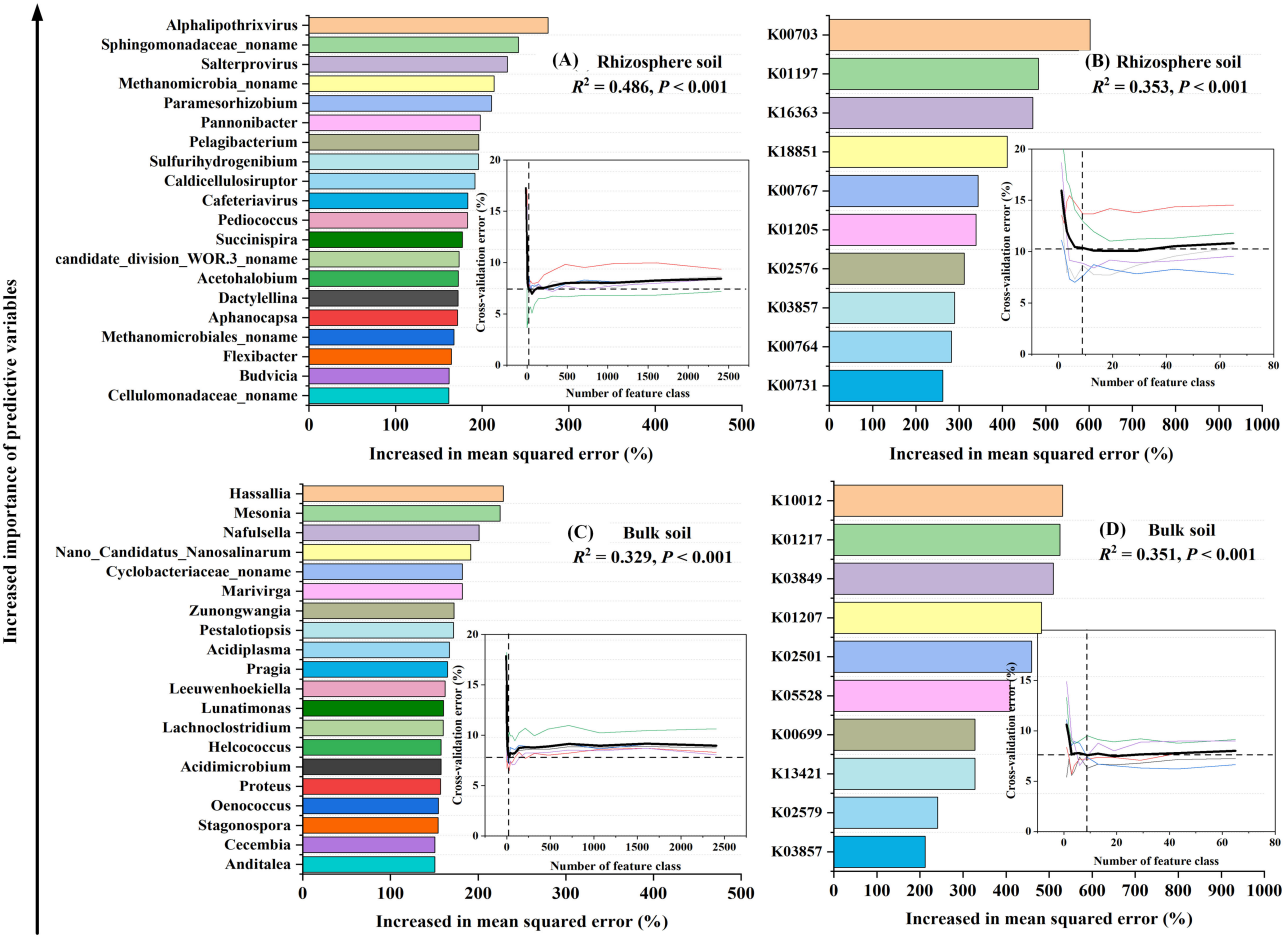
Random forest analysis identifying the significant (*P* < 0.05) microbial predictors of soil respiration components: the relative abundance of the soil microbial community at the genus level **(A, C)**, and the abundance of functional genes **(B, D)**. These functional genes were annotated according to the CAZy (carbohydrate active enzyme) database using metagenomic data derived from a subset of soil samples (**Supplementary Table S3**). Embedded in the figure are five 10-fold cross-validation results to assess the importance of microbial variables. When the cross-validation error is at the minimum (stable), the prediction model has the best performance. The importance of the variable is measured by the “percentage of increase of mean square error (Increase in MSE(%))” value in the random forest. Higher MSE% values mean more important variables. In the figure, the importance of microbial variables to model accuracy decreases in turn.

Utilizing a random forest model and five iterations of 10-fold cross-validation, we identified the optimal combination of 20 genera and 10 functional genes that encapsulate sample feature information (*P* < 0.05). These combinations accounted for variations in Rs components ranging from 0.329 to 0.486 (*P* < 0.001, **Figure 5**). The chosen carbon-degrading bacteria primarily consisted of bacterial communities, with certain selected genera and functional genes showing significant correlations with Rs components (*P* < 0.05, **Supplementary Table S4**).

The PLS-PM model revealed that soil properties and functional attributes exerted significant direct positive influences on both Ra and Rh (with Goodness-of-Fit values of 0.67 and 0.62, respectively), whereas taxonomic attributes did not show a statistically significant impact (**Figure 4**). Soil properties positively influenced root traits and functional attributes, indirectly affecting Ra through these pathways. Consequently, alterations in Rs components across environmental gradients were primarily achieved through modifications in root traits and microbial functional attributes.

## 4 Discussion

### 4.1 Relative contributions of Rh and Ra to Rs along the environmental gradient

Prior research indicates that Rs and soil physicochemical properties exhibit high variability within desert ecosystems (Wang et al., 2021b). Precisely categorizing observed Rs into various components and evaluating their environmental responsiveness is crucial for comprehending terrestrial ecosystem carbon balances (Chin et al., 2023). This study determined that Ra’s contribution to Rs ranged from 37% to 45% at the research site, aligning with previously reported ranges for desert ecosystems (13%-94%) (Zhao et al., 2011). However, these figures differ from those in other ecosystems (Cahoon et al., 2016; Huang et al., 2015) due to variations in site-specific conditions and measurement methodologies affecting Ra’s contribution to Rs (Hanson et al., 2000). Moreover, Rh accounted for over 50% of Rs in all three plots, clearly indicating Rh as the primary driver of Rs, corroborating earlier findings (Chen et al., 2015; Ma et al., 2019; Subke et al., 2006). Research in an alpine meadow on the Qinghai-Tibet Plateau revealed that warming decreased the Rh/Rs ratio, primarily attributed to minimal Rh variation and Ra augmentation induced by warming (Yan et al., 2022). Conversely, a study in a drier temperate evergreen broadleaf forest indicated that diminished rainfall resulted in a 40% reduction in root respiration, with no impact on Rh, thus elevating the Rh/Rs ratio (Hinko-Najera et al., 2015). Notably, this study discovered that environmental stress enhanced Rh’s relative contribution to Rs (**Table 1**), typically explained by Ra experiencing a more pronounced reduction under drought stress compared to Rh (Balogh, 2016; Li et al., 2013). To sum up, these findings suggest that root activities are more suppressed in arid desert ecosystems (Zhou et al., 2019), leading to a higher percentage of carbon emissions originating from soil microbes rather than root and rhizosphere respiration (Wang et al., 2019b). As global climate change and drought frequency rise (Knapp et al., 2015), future climates in arid deserts could significantly alter Rs through shifts in component intensities and proportions (Liu et al., 2017). Soil moisture might also play a crucial role in the carbon cycle during the growing seasons in arid regions (Kannenberg et al., 2024).

### 4.2 Changes in Ra driven by root traits and rhizosphere microorganisms

Consistent with the initial hypothesis, environmental stress can affect Ra by altering microbial activity and plant physiological structure (Vries et al., 2016). This study identified a diverse range of microorganisms, including bacteria, archaea, fungi, and viruses, in the rhizosphere (**Table 2**). Additionally, shifts in the microbial community’s composition and structure were observed across different environmental gradients (**Figure 3**; **Supplementary Figure S1**). This may be due to the fact that these microorganisms are adjacent to plant roots, and their combination can obtain nutrients for plants to promote plant growth and health (Abraham and Babalola, 2019). Some researchers have pointed out that environmental stress can lead to strong changes in rhizosphere microbial community structure and functional genes (Argaw et al., 2021; Naseem et al., 2018; Sanaullah et al., 2011; Segovia et al., 2013). Microorganisms that occupy the rhizosphere and promote plant growth and tolerance can adopt certain adaptive life history strategies to cope with changes in resource availability and abiotic conditions (Aslam et al., 2022; Ju et al., 2020; Li et al., 2022), thereby affecting Ra mediated by rhizosphere microorganisms. Studies have shown that life history strategies that focus on the interaction between microorganisms and the environment can help to link microbial ecology with ecosystem functions (Malik et al., 2019a). Character-based microbial life history strategies can be divided into three categories: high yield (Y), resource acquisition (A), and stress tolerance (S) (Malik et al., 2019a). In soils from river bank habitats to desert marginal habitats, the abundance of PL and CE genes involved in polysaccharides and carbohydrate esters decreased gradually, while the abundance of GH genes involved in soil organic C decomposition increased gradually (**Figure 4**), which meant that the microbial life history strategy changed from stress tolerance (S) to resource acquisition-stress tolerance combination (S-A) (Naseem et al., 2018). This is because with the increase of environmental stress, microorganisms not only need to increase their investment in extracellular polysaccharide (EPS) to cope with drought stress (Naseem et al., 2018) but also need to increase investment in extracellular enzyme production to alleviate resource constraints (Allison and Vitousek, 2005; Lipson, 2015). However, this strategic shift reduces metabolism in other physiological processes (e.g., Ra) due to increased metabolic investment in resource acquisition, typically resulting in slower cell growth rates (Malik et al., 2019a).

In addition, the random forest model identified a new list of key microbial classification and functional attributes (**Figure 5**). These microbial attributes were significantly correlated with Ra (*P* < 0.05, **Figure 5**), and they were important Ra predictors along the environmental gradient. Among them, Ra was significantly positively correlated with related genera in Firmicutes (e.g., *Caldicellulosiruptor*, *Pediococcus*, *Succinispira*, and *Acetohalobium*) and significantly negatively correlated with related genera in Proteobacteria (e.g., *Paramesorhizobium*, *Pannonibacter*, and *Pelagibacterium*) (**Supplementary Table S4**). An increasing number of studies have shown that archaea exist in various environments on Earth (Flemming and Wuertz, 2019; Shu and Huang, 2022), and these microorganisms play important roles in biochemical processes such as carbon fixation, methane oxidation, methane production, and organic matter degradation (Adam et al., 2017). Although archaea are considered to be rare members of the microbial biosphere, some archaea have been shown to be major members of ecosystems (Spang et al., 2017). Among them, Methanobacteriales and Methanococcales, as methanogenic archaea, conduct the anaerobic fermentation of inorganic or organic compounds to convert them into methane and carbon dioxide (Evans et al., 2019; Kurth et al., 2021). In this study, a positive correlation was detected between the related genera of Euryarchaeota (*Methanomicrobia noname* and *Methanomicrobiales noname*) and Ra (**Supplementary Table S4**), which is consistent with previous research results (Zhang and Lv, 2020). This result indicates that archaea are also involved in the regulation of rhizosphere soil carbon metabolism, but the current research is still in its infancy. In the future, next-generation omics techniques (such as metatranscriptomics and metaproteomics) are needed to verify the ecological functions of these archaea, thereby increasing the understanding of carbon metabolism in arid desert ecosystems.

**Figure 6:**
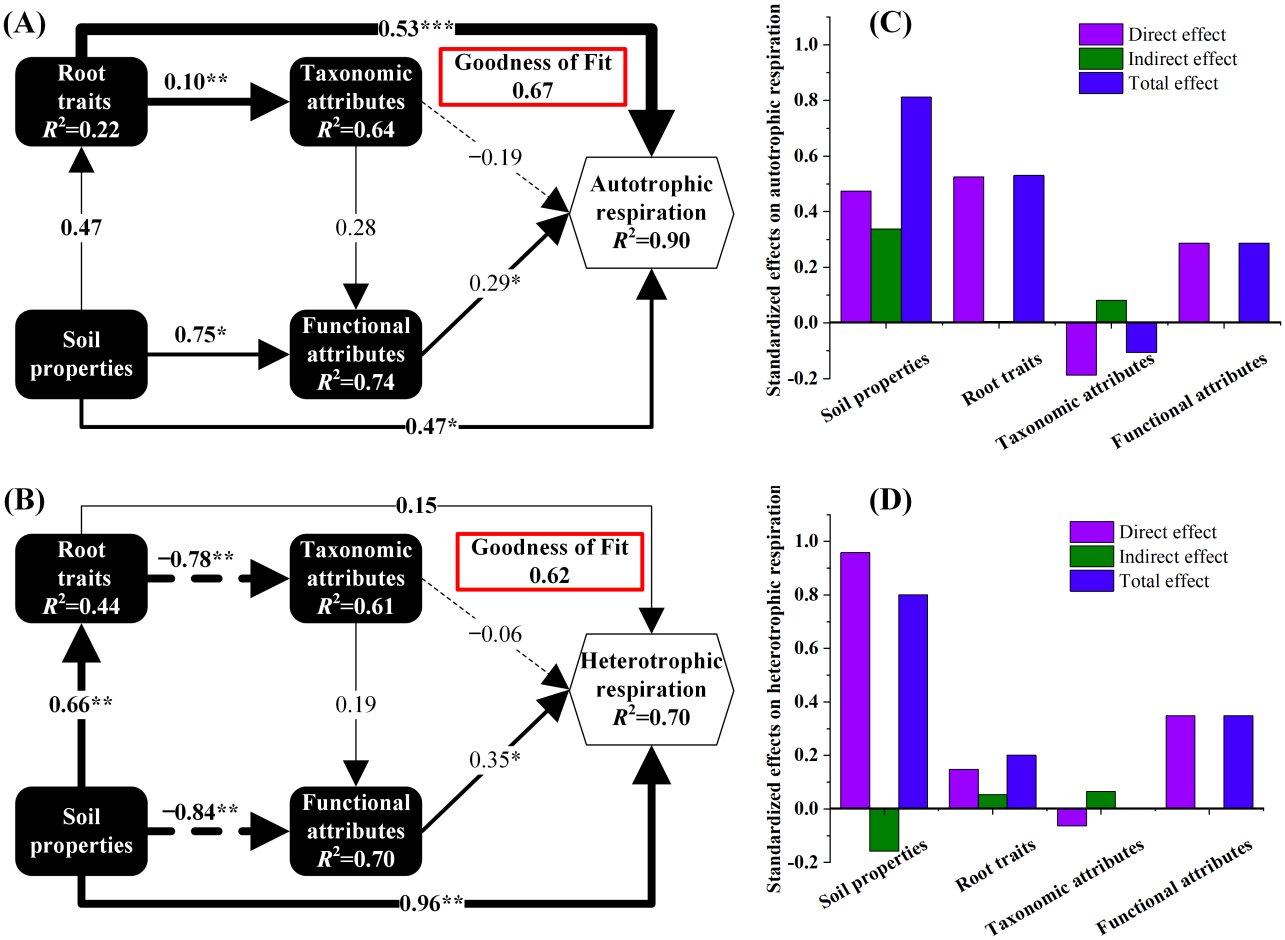
Partial least squares path modeling (PLS-PM) for determining the effects of soil properties, root traits, taxonomic attributes, and functional attributes on soil respiration components **(A, B)** and standardized path coefficients **(C, D)**. Arrow widths describe the magnitude of the path coefficients, and solid and dashed arrows indicate positive and negative effects, respectively. *P < 0.05, **P < 0.01. Microbial classification attributes and functional attributes are represented by the first and second axes of non-metric multidimensional scale ranking (NMDS) based on the Bray–Curtis distance, respectively. More information about NMDS is provided in **Supplementary Figure S3**.

There is evidence that different microorganisms can perform similar metabolic functions due to horizontal gene transfer between microorganisms, and their communities usually exhibit high ‘functional redundancy’ (Louca et al., 2016). The research framework based on microbial taxonomy does not necessarily reflect the significant impact of species diversity reduction or community species composition changes on their mediated ecological processes or the response to environmental changes (Green et al., 2008). Interestingly, it was found that soil properties, root traits, and microbial functional attributes were closely related to Ra (**Figure 6**), which provides direct evidence that soil function is driven by soil properties, root traits, and microbial functional genes (Spitzer et al., 2021; Trivedi et al., 2016), and suggests that microbial functional attributes rather than taxonomic attributes may provide a more effective link between microorganisms and Ra (Chen et al., 2020). The gene abundances of starch synthase (K00703), phosphatidylinositol glycan A (K03857) encoding GTs and UDP-3-O-[3-hydroxymyristoyl] N-acetylglucosamine deacetylase (K16363), and heparan sulfate N-deacetylase/N-sulfotransferase1 (K02576) encoding CEs were significantly positively correlated with Ra (*P* < 0.05, **Supplementary Table S4**). The CAZy annotation results showed that these genes were key genes in carbohydrate metabolism and lipid metabolism (Kanehisa et al., 2015). Therefore, paying attention to these genes may be of great significance for understanding the soil carbon cycle in desert ecosystems, and these genes may be involved in the related processes of microbial respiration and carbon metabolism in rhizosphere soil.

Environmental changes, altering soil water conditions and nutrient availability, directly or indirectly affect Ra (Bloor and Bardgett, 2012; Tang et al., 2020b). Typically, in natural environments, low SWC leads to high ST, restricting root and microbial metabolism (Liu et al., 2018; Luo and Zhou, 2006), and consequently suppressing root and rhizosphere respiration (Jarvi and Burton, 2013). Brunner et al. (2015) noted that during drought, tree species allocate significant carbon to defense and storage, like woody tissues, instead of acquiring external resources. This strategy enhances plant resilience to environmental stress, aligning with current findings. The study observed that SRA rises with drought stress, suggesting that more carbon is directed towards increasing underground biomass rather than respiration, significantly lowering Ra (**Figure 2**; **Table 1**). This can be attributed to the fact that under drought stress conditions, plants can preferentially allocate more carbon to root storage pools and osmotic adjustment, while the proportion used for their own metabolic consumption is relatively reduced, and the decline in Ra is due to the reduction of metabolic activity, which improves their adaptability (Hasibeder et al., 2014). In addition, because phosphorus is the main component of adenosine triphosphate (ATP), a product of root respiration, the decrease of phosphorus content in plant tissues inhibits the decrease of ATP synthesis and thus limits Ra (Jarvi and Burton, 2017). Overall, the results of this study highlight that in order to provide accurate information on future belowground carbon partitioning and carbon cycling, it may be effective to evaluate Ra patterns associated with soil properties, root traits, and rhizosphere microorganisms (especially functional genes) (Jia et al., 2013).

### 4.3 Revealing the subsurface driving factors of Rh

The bare soil environment is a typical oligotrophic environment, in which most of the microorganisms survive and grow slowly in the soil in an adaptive oligotrophic metabolic model (Chen et al., 2021). Evidence suggests that numerous oligotrophic microorganisms, such as actinomycetes, facilitate soil organic matter decomposition by aiding in the breakdown of challenging compounds like cellulose and lignin (Kanokratana et al., 2010; Li et al., 2016). Our findings reveal differences in microbial community structure and composition between the rhizosphere and bulk soils (**Table 2**). Bulk soil microbiota was predominantly bacterial, with actinomycetes showing the greatest relative abundance (**Figure 3**; **Supplementary Figure S1**), aligning with prior research on alpine peatland microbial communities (Kang et al., 2022). This could stem from the selective pressure of the oligotrophic bare environment, favoring actinomycete groups adapted to prolonged oligotrophic metabolic conditions (Zhang et al., 2018b). Research indicates that over 90% of enzymes responsible for carbohydrate degradation originate primarily from Firmicutes and Bacteroidetes (Gong et al., 2020). We observed that environmental stress decreased the relative abundance of Bacteroidetes in bulk soil (**Figure 3**), potentially accounting for the reduction in Rh from river bank to desert margin. Furthermore, our analysis identified 20 microbial genera associated with Rh, including nine from Bacteroidetes. Consequently, we propose that Bacteroidetes could serve as a valuable biological indicator for estimating soil Rh variations across different environmental gradients.

It is worth noting that in the present study, a complex gene pool composed of diverse enzyme families was identified in bulk soil (**Supplementary Figure S2**), which can provide a variety of degradation capabilities for the utilization of various carbohydrate substrates (Gong et al., 2020). Compared with rhizosphere soil, bulk soil had a higher abundance of GH carbohydrate active genes (**Figure 4**), and these enzymes were assigned to 109 GH families (**Supplementary Figure S2**). This is because in bulk soils with low resource availability, microorganisms increase investment in extracellular enzyme production to break down complex resources (Allison et al., 2010), resulting in richer GH genes and higher GH enzyme activity (Malik et al., 2019b). In addition, beta-N-acetylhexosaminidase (K00703) and L-iduronidase (K01217) genes encoding GHs exhibited positive effects on Rh (**Supplementary Table S4**), which is consistent with other research results that these gene predictors are significantly correlated with soil C metabolic functioning (Yang et al., 2021). Surprisingly, the present study found that phosphatidylinositol glycan A (K03857) could also explain the change of Rh (**Supplementary Table S4**), which was consistent with Ra. Phosphatidylinositol glycan A is a gene encoding the catalytic subunit of GPI-N-glucosamine acetate transferase and a key enzyme in the biosynthesis of glycosylphosphatidylinositol (GPI) (Miyata et al., 1993). Therefore, we suggest that identifying this key functional gene is essential if microbial communities are to be used to predict patterns of changes in respiratory components in different environments. Although previous studies have thoroughly demonstrated that Rh, as a complex process, is closely related to the microbial community composition and soil properties (Whitaker et al., 2014; Zhang et al., 2021), this study also evaluated the effects of microbial functional attributes on Rh on the basis of previous studies. In PLS-PM, the relationship between Rh and microbial functional attributes was superior to the taxonomic attributes (**Figure 6**), which further confirmed the importance of incorporating microbial functional genes into Rs models (Liu et al., 2019; Xu et al., 2018; Yang et al., 2021).

Among the three methods for distinguishing Ra and Rh, the root exclusion method stands out as the simplest and most dependable for extensive *in-situ* separation of Rs components (Borden et al., 2021; Zhao et al., 2011). Root exclusion methods commonly involve root removal, trenching, and gap analysis (Chin et al., 2023). To address the inherent limitations of the root exclusion method (Hopkins et al., 2013; Zhao et al., 2011) and given the ease of measuring multiple sampling points, this study integrated trenching and gap analysis to separate various Rs components. Despite performing the initial Rs measurement after 10 months of trenching to mitigate soil disturbance and dead root decomposition effects (Chin et al., 2023; Schiedung et al., 2023), it’s important to acknowledge that this approach carries uncertainties. The fundamental premise of the root exclusion method is that Rh stays constant post-root and associated root exudate removal (Chin et al., 2023; Zhao et al., 2011). It’s noteworthy that even though bare soil lacks inputs of readily decomposable organic matter like root exudates and plant residues, microbes can influence Rh by breaking down more recalcitrant soil carbon pools (Tian et al., 2024). To sum up, the root exclusion method alters microbial substrate availability by decreasing root exudates rich in labile carbon, potentially impacting the ‘priming effect’ on soil carbon mineralization (Chin et al., 2023). This influences soil organic carbon decomposition rates, possibly causing variations in Rh estimates (Classen et al., 2015). Obviously, future studies are required to investigate how the priming effect impacts Rh under prolonged root exclusion, aiming to resolve existing uncertainties.

## 5 Conclusion

In summary, our research represents an important step in unraveling the complex relationships between soil microbial communities, soil properties, root traits, and Rs components within the unique context of the desert-oasis ecotone in arid regions. Through a comprehensive field investigation and advanced metagenomic analysis, we have provided novel insights into the factors governing Rs and its autotrophic and heterotrophic components.

**Figure 7:**
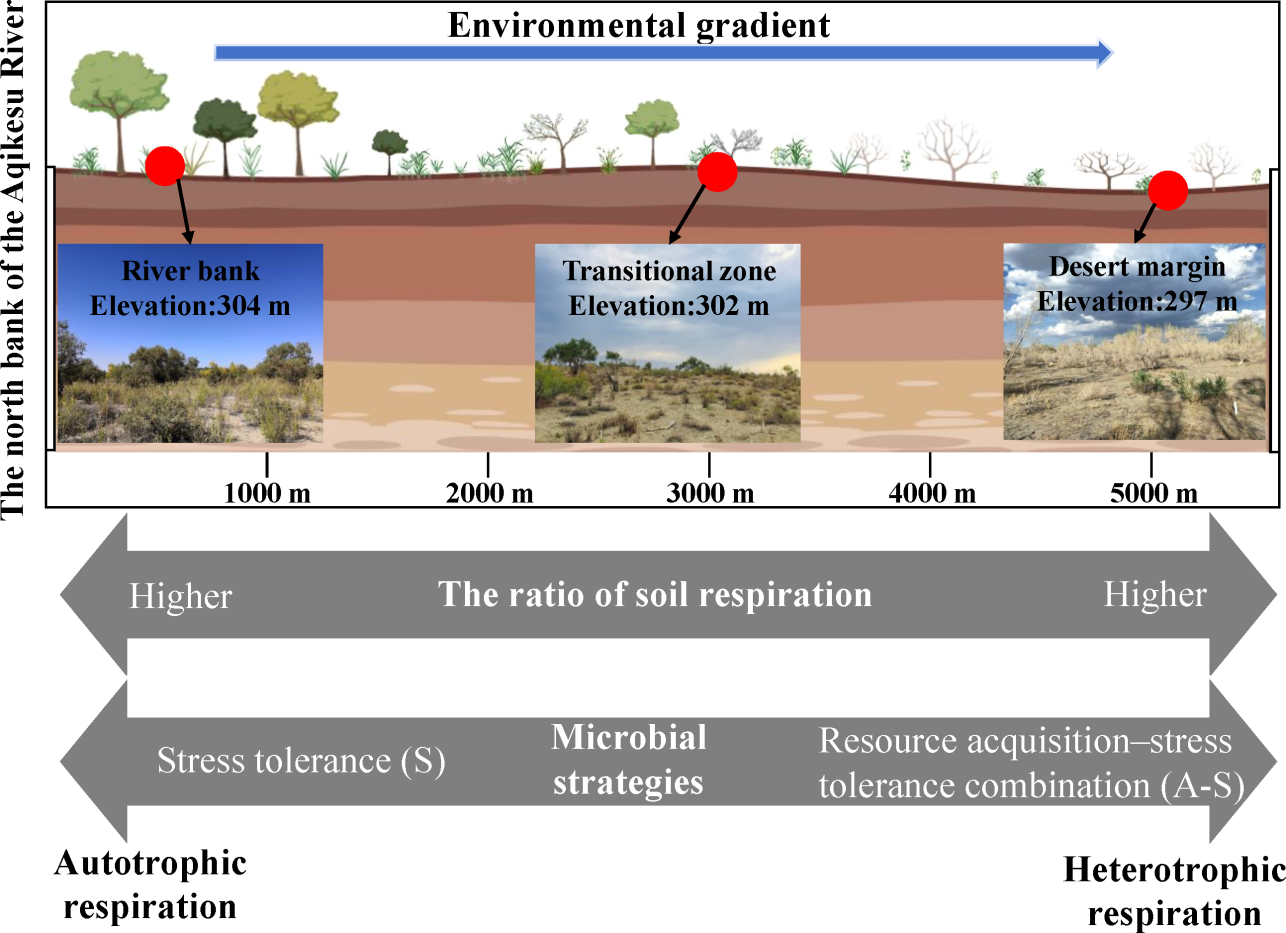
Underlying mechanisms of abiotic and biotic drivers on soil respiration components and their changes in a desert-oasis ecotone in an arid region.

Our findings clearly demonstrate that environmental stress, manifested as a gradient from the relatively more favorable river bank to the harsher desert margin, exerts a significant negative impact on Rs and its constituent parts. This stress not only affects the biotic drivers, such as root and microbial traits, but also the abiotic drivers, including soil microclimate and nutrient levels (**Figure 7**). Importantly, we have discovered that the components of Rs are more responsive to microbial functional genes than to microbial taxonomic diversity. This indicates that the balance between microbial metabolic expenditure and the presence of carbon-degrading genes plays a crucial role in determining the contribution of microbial activities to Rs components.

These results have important implications for future research on the carbon cycle in desert ecosystems. We emphasize the need to distinguish between the autotrophic and heterotrophic components of Rs when evaluating their responses to environmental changes. Moreover, we recommend the utilization of traits, including microbial functional genes, root traits, and chemical properties, as essential regulatory elements in Rs modeling. This approach will enhance the precision of predicting soil carbon emissions and carbon cycling in these arid regions, thereby providing a more accurate understanding of the role of desert ecosystems in the global carbon cycle. Our study thus serves as a foundation for further investigations aimed at developing effective strategies for carbon management and ecosystem conservation in arid and semi-arid regions.

## Data Availability

The original contributions presented in the study are included in the article/**Supplementary Material**. Further inquiries can be directed to the corresponding authors.
