# Supplementary material for: Impacts of plant root traits and microbial functional attributes on soil respiration components in the desert-oasis ecotone
Source: Front Plant Sci. 2025 Feb 11;16:1511277. doi: 10.3389/fpls.2025.1511277 (PMC11850576; doi:10.3389/fpls.2025.1511277)
Supplement: Supplementary file 1 [file DataSheet1.docx]

**Supplementary material**

# Supplementary Figures and Tables

1.1 Supplementary Tables

Supplementary Table S1

Soil properties in three habitat types. Values represent the mean ± SE over the experimental period (May–Aug.)

| Plot | River bank | | Transitional zone | | Desert margin | |
| --- | --- | --- | --- | --- | --- | --- |
|  | Rhizosphere soil | Bulk soil | Rhizosphere soil | Bulk soil | Rhizosphere soil | Bulk soil |
| Soil temperature  (°C) | 19.16 ± 1.08  Ba | 19.39 ± 0.55  Ca | 21.01 ± 2.30  ABa | 22.58 ± 0.40 Ba | 22.06 ± 2.37  Aa | 23.47 ± 0.47  Aa |
| Soil water content  (%) | 16.64 ± 2.42  Aa | 15.59 ± 2.26 Aa | 3.54 ± 1.56  Ba | 3.71 ± 0.28  Ba | 3.28 ± 0.58  Ba | 2.62 ± 1.24  Ba |
| Soil total phosphorus  (g kg^−1^) | 0.89 ± 0.13  Aa | 0.80 ±0.04  Aa | 0.70 ± 0.09  Ba | 0.71 ± 0.18  Aa | 0.62 ± 0.08  Ba | 0.40 ± 0.04  Bb |
| Soil available phosphorus  (g kg^−1^) | 71.56 ± 18.57  Ab | 95.54 ± 13.94  Aa | 48.15 ± 18.00  Ba | 14.38± 2.21  Bb | 43.28 ± 9.38  Ba | 13.81± 1.29  Bb |
| Soil ammonium nitrogen  (g kg^−1^) | 10.28 ± 3.74  Aa | 3.45 ± 0.52 Ab | 8.64 ± 1.92  Aa | 2.97 ± 0.34  Ab | 9.78 ± 2.14  Aa | 3.80 ± 1.16  Ab |
| Soil nitrate nitrogen  (g kg^−1^) | 36.07 ± 10.60  Aa | 55.13 ± 26.89  Aa | 45.63 ± 14.31  Aa | 25.87 ± 20.00 Ba | 36.06 ± 10.78  Aa | 8.37 ± 3.01  Bb |
| Total nitrogen content  (g kg^−1^) | 0.95 ± 0.13  Aa | 0.61 ± 0.07 Ab | 0.75 ± 0.11  Ba | 0.48 ± 0.08  Bb | 0.59 ± 0.13  Ba | 0.36 ± 0.05  Cb |
| Soil organic carbon content  (g kg^−1^) | 20.39 ± 3.91  Aa | 10.29 ± 2.39 Ab | 14.81 ± 3.57  ABa | 8.25 ± 5.96  Aa | 13.18 ± 6.19  Ba | 1.89 ± 0.30  Bb |
| Soil salinity content  (g kg^−1^) | 6.18 ± 1.95  Aa | 8.31 ± 2.46 Aa | 5.57 ± 1.55  Aa | 4.27 ± 2.10  Ba | 5.01 ± 1.45  Aa | 2.27 ± 0.58  Bb |
| Soil pH | 8.25 ± 0.15  Aa | 8.07 ± 0.16 Aa | 7.88 ± 0.12  Ba | 8.02 ± 0.41  Aa | 7.50 ± 0.15  Ca | 7.41 ± 0.25  Ba |
| Clay (%) | 2.21 ± 0.30  Aa | 2.27 ± 0.90 Aa | 0.97 ± 0.42  Ba | 4.24 ± 0.95  Aa | 0.19 ± 0.27  Ca | 0.02 ± 0.05  Ba |
| Silt (%) | 24.87 ± 1.11  Aa | 27.50 ± 5.16 Aa | 8.95 ± 4.00  Bb | 19.70 ± 8.06  Ba | 5.41 ± 2.98  Ba | 2.86 ± 1.08  Ca |
| Sand (%) | 72.92 ± 1.32  Ba | 70.24± 5.98 Ba | 90.09 ± 4.12  Aa | 76.07 ± 8.72  Ba | 94.4 ± 3.23  Aa | 97.10 ± 1.07  Aa |

Supplementary Table S2

Statistics of assembly results. Clean reads: data after quality control; No.: number of contig obtained by splicing; Max Len: the longest contig length; Total Len: total length of all contig; Average Len: average length of contig; GC content: GC content of conFigure R1–R6, T1–T6, and D1–D6 are rhizosphere soils; R7–R12, T7–T9, and D7–D12 are bulk soils.

| Sample | Clean reads | No. | Max Len | Total Len | Average Len | GC content |
| --- | --- | --- | --- | --- | --- | --- |
| R1 | 57506238 | 565037 | 118 621 | 537527419 | 951.31 | 65.29% |
| R2 | 46217770 | 456029 | 111 159 | 420386751 | 921.84 | 65.34% |
| R3 | 48844262 | 481528 | 108 788 | 450796112 | 936.18 | 65.42% |
| R4 | 69915120 | 694356 | 433 565 | 794124808 | 1143.69 | 62.09% |
| R5 | 60413702 | 537316 | 160 436 | 570539043 | 1061.83 | 63.25% |
| R6 | 48677766 | 421192 | 206 975 | 433343983 | 1028.85 | 65.57% |
| T1 | 40110172 | 387152 | 88 174 | 344828684 | 890.68 | 65.50% |
| T2 | 48850174 | 478723 | 72 037 | 441222648 | 921.67 | 65.47% |
| T3 | 45124056 | 449813 | 64 387 | 411697447 | 915.26 | 65.39% |
| T4 | 58458514 | 634036 | 289 405 | 691220921 | 1090.19 | 62.84% |
| T5 | 57432662 | 593739 | 308 707 | 615681104 | 1036.96 | 63.04% |
| T6 | 44978628 | 498120 | 358 646 | 508551696 | 1020.94 | 63.53% |
| D1 | 48764440 | 507441 | 319 290 | 513049796 | 1011.05 | 63.35% |
| D2 | 54618982 | 587019 | 310 289 | 588329336 | 1002.23 | 63.94% |
| D3 | 41367508 | 436674 | 338 779 | 424328585 | 971.73 | 63.57% |
| D4 | 56294738 | 573066 | 268 735 | 550326726 | 960.32 | 64.95% |
| D5 | 49478494 | 538845 | 174 610 | 510166463 | 946.78 | 65.03% |
| D6 | 44324736 | 465617 | 99 483 | 431842454 | 927.46 | 65.03% |
| R7 | 85442756 | 692416 | 242 230 | 711815678 | 1028.02 | 66.48% |
| R8 | 67693094 | 545031 | 304 014 | 585744706 | 1074.7 | 65.11% |
| R9 | 41388406 | 428690 | 50 022 | 427312052 | 996.79 | 63.75% |
| R10 | 55662648 | 541576 | 170 739 | 572053230 | 1056.28 | 59.24% |
| R11 | 72456672 | 584363 | 131 825 | 588960754 | 1007.87 | 67.19% |
| R12 | 72297178 | 582807 | 189 303 | 620110042 | 1064.01 | 65.92% |
| T7 | 67715652 | 526719 | 183 897 | 571906483 | 1085.79 | 64.83% |
| T8 | 67563592 | 502393 | 211 861 | 557710021 | 1110.11 | 66.34% |
| T9 | 54311530 | 459077 | 120 322 | 483122457 | 1052.38 | 66.29% |
| D7 | 63897786 | 524990 | 74 190 | 445647482 | 848.87 | 67.48% |
| D8 | 67161870 | 591779 | 72 656 | 520210879 | 879.06 | 66.16% |
| D9 | 73318674 | 634367 | 581 555 | 569128571 | 897.16 | 67.16% |
| D10 | 66253468 | 498752 | 99 654 | 432929115 | 868.02 | 67.39% |
| D11 | 67983838 | 597667 | 43 040 | 499401420 | 835.58 | 67.65% |
| D12 | 67771482 | 573886 | 264 610 | 558756848 | 973.64 | 67.04% |

Supplementary Table S3

The ID and definition of the predicted functional genes annotated according to the CAZy (carbohydrate active enzyme) database using metagenomic data derived from a subset of soil samples.

| **KO**  **ID** | **Definition** |
| --- | --- |
| K00635 | diacylglycerol O-acyltransferase [EC:2.3.1.20] |
| K18851 | fbp; diacylglycerol O-acyltransferase / trehalose O-mycolyltransferase [EC:2.3.1.20 2.3.1.122] |
| K03476 | ulaG; L-ascorbate 6-phosphate lactonase [EC:3.1.1.-] |
| K02579 | NDST4; heparan sulfate N-deacetylase/N-sulfotransferase NDST4 [EC:3.1.1.- 2.8.2.-] |
| K02576 | NDST1; heparan sulfate N-deacetylase/N-sulfotransferase NDST1 [EC:3.1.1.- 2.8.2.-] |
| K01046 | triacylglycerol lipase [EC:3.1.1.3] |
| K01051 | pectinesterase [EC:3.1.1.11] |
| K01184 | polygalacturonase [EC:3.2.1.15] |
| K13014 | arnD; undecaprenyl phosphate-alpha-L-ara4FN deformylase [EC:3.5.1.-] |
| K02535 | lpxC; UDP-3-O-[3-hydroxymyristoyl] N-acetylglucosamine deacetylase [EC:3.5.1.108] |
| K01195 | uidA, GUSB; beta-glucuronidase [EC:3.2.1.31] |
| K01197 | hya; hyaluronoglucosaminidase [EC:3.2.1.35] |
| K01205 | NAGLU; alpha-N-acetylglucosaminidase [EC:3.2.1.50] |
| K01207 | nagZ; beta-N-acetylhexosaminidase [EC:3.2.1.52] |
| K01213 | galacturan 1,4-alpha-galacturonidase [EC:3.2.1.67] |
| K01217 | IDUA; L-iduronidase [EC:3.2.1.76] |
| K01230 | MAN1; mannosyl-oligosaccharide alpha-1,2-mannosidase [EC:3.2.1.113] |
| K01236 | treZ, glgZ; maltooligosyltrehalose trehalohydrolase [EC:3.2.1.141] |
| K02438 | treX, glgX; glycogen operon protein [EC:3.2.1.-] |
| K12373 | HEXA_B; hexosaminidase [EC:3.2.1.52] |
| K00700 | glgB; 1,4-alpha-glucan branching enzyme [EC:2.4.1.18] |
| K00720 | UGCG; ceramide glucosyltransferase [EC:2.4.1.80] |
| K00731 | C1GALT1; glycoprotein-N-acetylgalactosamine 3-beta-galactosyltransferase [EC:2.4.1.122] |
| K00733 | B4GALT7; xylosylprotein 4-beta-galactosyltransferase [EC:2.4.1.133] |
| K00748 | lpxB; lipid-A-disaccharide synthase [EC:2.4.1.182] |
| K00762 | pyrE; orotate phosphoribosyltransferase [EC:2.4.2.10] |
| K00764 | purF, PPAT; amidophosphoribosyltransferase [EC:2.4.2.14] |
| K00766 | trpD; anthranilate phosphoribosyltransferase [EC:2.4.2.18] |
| K02841 | waaC, rfaC; heptosyltransferase I [EC:2.4.-.-] |
| K02849 | waaQ, rfaQ; heptosyltransferase III [EC:2.4.-.-] |
| K03842 | ALG1; beta-1,4-mannosyltransferase [EC:2.4.1.142] |
| K05528 | OCH1; alpha 1,6-mannosyltransferase [EC:2.4.1.232] |
| K05535 | MNN2; alpha 1,2-mannosyltransferase [EC:2.4.1.-] |
| K07151 | STT3; dolichyl-diphosphooligosaccharide--protein glycosyltransferase [EC:2.4.99.18] |
| K16148 | glgA; starch synthase [EC:2.4.1.21] |
| K16149 | K16149; 1,4-alpha-glucan branching enzyme [EC:2.4.1.18] |
| K00699 | UGT; glucuronosyltransferase [EC:2.4.1.17] |
| K00710 | GALNT; polypeptide N-acetylgalactosaminyltransferase [EC:2.4.1.41] |
| K00729 | ALG5; dolichyl-phosphate beta-glucosyltransferase [EC:2.4.1.117] |
| K00737 | MGAT3; beta-1,4-mannosyl-glycoprotein beta-1,4-N-acetylglucosaminyltransferase [EC:2.4.1.144] |
| K02527 | kdtA, waaA; 3-deoxy-D-manno-octulosonic-acid transferase [EC:2.4.99.12 2.4.99.13 2.4.99.14 2.4.99.15] |
| K02844 | waaG, rfaG; UDP-glucose:(heptosyl)LPS alpha-1,3-glucosyltransferase [EC:2.4.1.-] |
| K02847 | waaL, rfaL; O-antigen ligase [EC:2.4.1.-] |
| K03849 | ALG8; alpha-1,3-glucosyltransferase [EC:2.4.1.265] |
| K03857 | PIGA, GPI3; phosphatidylinositol glycan, class A [EC:2.4.1.198] |
| K05286 | PIGB; phosphatidylinositol glycan, class B [EC:2.4.1.-] |
| K05529 | MNN9; mannan polymerase complexes MNN9 subunit [EC:2.4.1.-] |
| K07432 | ALG13; beta-1,4-N-acetylglucosaminyltransferase [EC:2.4.1.141] |
| K07542 | PIGV; phosphatidylinositol glycan, class V [EC:2.4.1.-] |
| K10012 | arnC, pmrF; undecaprenyl-phosphate 4-deoxy-4-formamido-L-arabinose transferase [EC:2.4.2.53] |
| K00688 | glgP, PYG; starch phosphorylase [EC:2.4.1.1] |
| K00703 | glgA; starch synthase [EC:2.4.1.21] |
| K00765 | hisG; ATP phosphoribosyltransferase [EC:2.4.2.17] |
| K00767 | nadC, QPRT; nicotinate-nucleotide pyrophosphorylase (carboxylating) [EC:2.4.2.19] |
| K00768 | cobU, cobT; nicotinate-nucleotide--dimethylbenzimidazole phosphoribosyltransferase [EC:2.4.2.21] |
| K00772 | mtaP, MTAP; 5'-methylthioadenosine phosphorylase [EC:2.4.2.28] |
| K01663 | HIS7; glutamine amidotransferase / cyclase [EC:2.4.2.- 4.1.3.-] |
| K02501 | hisH; glutamine amidotransferase [EC:2.4.2.-] |
| K10798 | PARP; poly [ADP-ribose] polymerase [EC:2.4.2.30] |
| K13421 | UMPS; uridine monophosphate synthetase [EC:2.4.2.10 4.1.1.23] |
| K14368 | eryCIII; 3-alpha-mycarosylerythronolide B desosaminyl transferase [EC:2.4.1.278] |
| K14375 | aveBI; dTDP-L-oleandrosyltransferase [EC:2.4.1.-] |
| K20430 | acbS; glycosyltransferase AcbS [EC:2.4.-.-] |
| K20438 | valG; glycosyltransferase ValG [EC:2.4.-.-] |
| K16363 | lpxC-fabZ; UDP-3-O-[3-hydroxymyristoyl] N-acetylglucosamine deacetylase / 3-hydroxyacyl-[acyl-carrier-protein] dehydratase [EC:3.5.1.108 4.2.1.59] |

Supplementary Table S4

Spearman correlation coefficients of the relative abundances of microbial taxonomic attributes (community composition) and functional attributes (functional genes) determined using random forest models with autotrophic respiration and heterotrophic respiration. P-values below 0.05 are shown in bold. Relevant information on the CAZy (carbohydrate active enzyme) database is available in Supplementary Table S3.

| Variable | Rhizosphere soil | | | Bulk soil | | |
| --- | --- | --- | --- | --- | --- | --- |
|  | Microbial | Correlation coefficients | *P* values | Microbial | Correlation coefficients | *P* values |
| Taxonomic attributes | Alphalipothrixvirus | **0.569** | **0.014** | Hassallia | **0.561** | **0.03** |
|  | Sphingomonadaceae_noname | −0.42 | 0.083 | Mesonia | **0.761** | **0.001** |
|  | Salterprovirus | **0.585** | **0.011** | Nafulsella | **0.593** | **0.02** |
|  | Methanomicrobia_noname | **0.581** | **0.011** | Nano_Candidatus_  Nanosalinarum | −0.5 | 0.058 |
|  | Paramesorhizobium | **−0.486** | **0.041** | Cyclobacteriaceae_noname | **0.724** | **0.002** |
|  | Pannonibacter | **−0.891** | **< 0.001** | Marivirga | **0.657** | **0.008** |
|  | Pelagibacterium | **−0.647** | **0.004** | Zunongwangia | **0.757** | **0.001** |
|  | Sulfurihydrogenibium | **0.684** | **0.002** | Pestalotiopsis | 0.489 | 0.064 |
|  | Caldicellulosiruptor | **0.474** | **0.047** | Acidiplasma | **−0.807** | **< 0.001** |
|  | Cafeteriavirus | **0.812** | **< 0.001** | Pragia | **0.679** | **0.005** |
|  | Pediococcus | **0.521** | **0.027** | Leeuwenhoekiella | **0.729** | **0.002** |
|  | Succinispira | **0.472** | **0.048** | Lunatimonas | **0.714** | **0.003** |
|  | candidate_division_WOR-3_noname | **0.548** | **0.019** | Lachnoclostridium | **−0.686** | **0.005** |
|  | Acetohalobium | **0.484** | **0.042** | Helcococcus | **0.739** | **0.002** |
|  | Dactylellina | **0.527** | **0.025** | Acidimicrobium | **−0.664** | **0.007** |
|  | Aphanocapsa | 0.408 | 0.093 | Proteus | **0.768** | **0.001** |
|  | Methanomicrobiales_noname | **0.69** | **0.002** | Oenococcus | **−0.568** | **0.02**7 |
|  | Flexibacter | 0.346 | 0.16 | Stagonospora | −0.496 | 0.06 |
|  | Budvicia | −0.063 | 0.804 | Cecembia | **0.729** | **0.002** |
|  | Cellulomonadaceae_noname | **−0.495** | **0.047** | Anditalea | **0.714** | **0.003** |
| Functional attributes | K00703 | **0.577** | **0.12** | K10012 | **−0.529** | **0.043** |
|  | K01197 | **−0.651** | **0.003** | K01217 | **0.738** | **0.002** |
|  | K16363 | **0.562** | **0.015** | K03849 | **0.843** | **0.001** |
|  | K18851 | −0.463 | 0.053 | K01207 | **0.582** | **0.023** |
|  | K00767 | 0.311 | 0.21 | K02501 | **0.704** | **0.003** |
|  | K01205 | −0.34 | 0.168 | K05528 | **0.694** | **0.004** |
|  | K02576 | **0.546** | **0.019** | K00699 | **0.599** | **0.018** |
|  | K03857 | **0.68** | **0.002** | K13421 | **−0.764** | **0.001** |
|  | K00764 | −0.401 | 0.099 | K02579 | **0.707** | **0.003** |
|  | K00731 | **−0.583** | **0.011** | K03857 | **0.63** | **0.12** |

**1.2 Supplementary Figures**


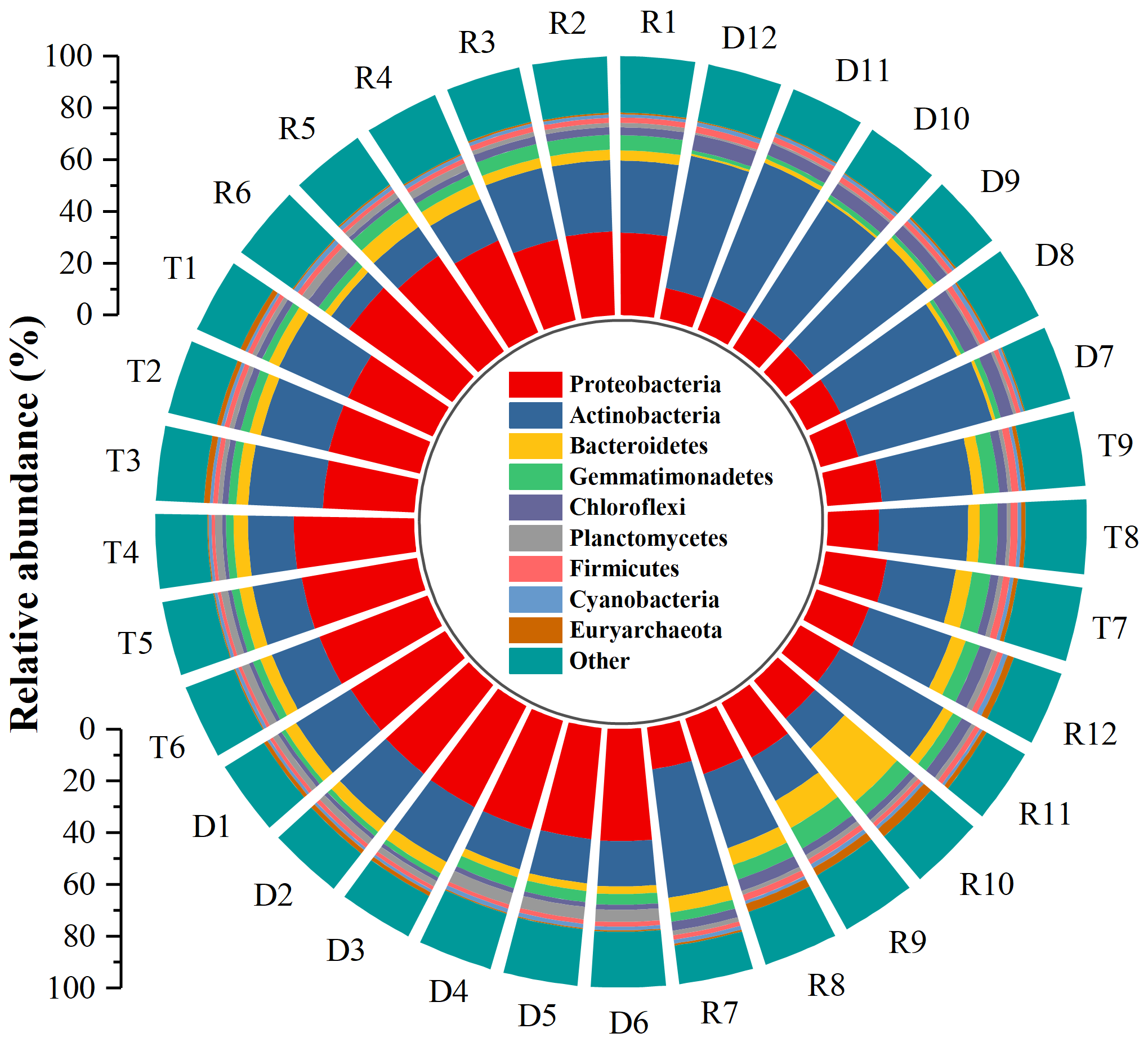


Supplementary Figure S1. Community composition and relative abundances of rhizosphere and bulk soil microbial communities at the phylum level. R1–R6, T1–T6, and D1–D6 are rhizosphere soils. R7–R12, T7–T9, and D7–D12 are bulk soils.


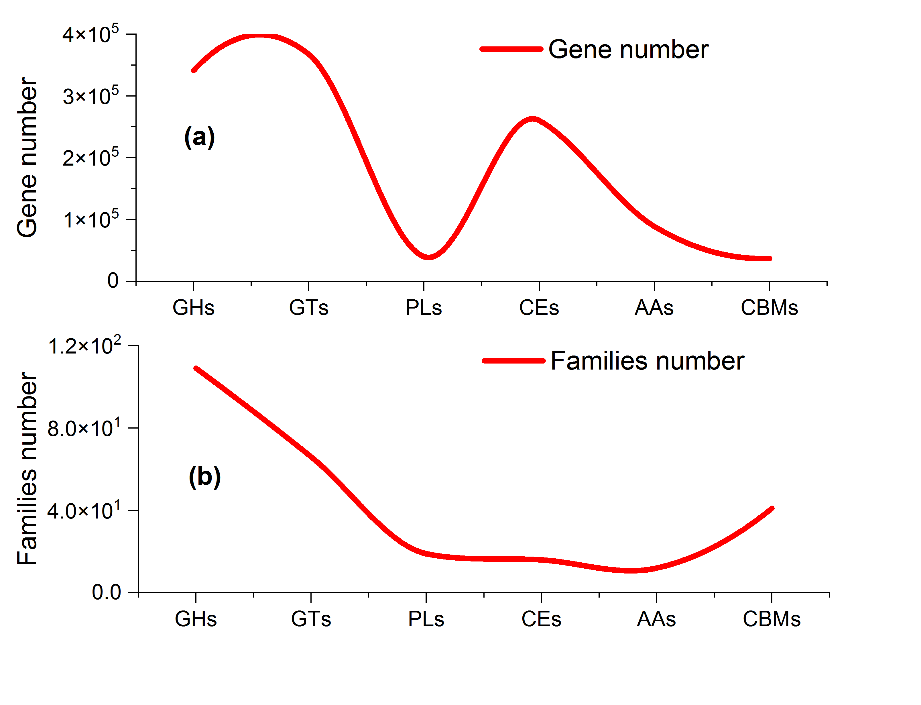


Supplementary Figure S2. Classification statistics obtained from the CAZy (Carbohydrate-Active enZYmes) database. Glycoside hydrolases (GHs), glycosyl transferases (GTs), polysaccharide lyases (PLs), carbohydrate esterases (CEs), auxiliary activities (AAs), carbohydrate binding modules (CBMs)*.*

**
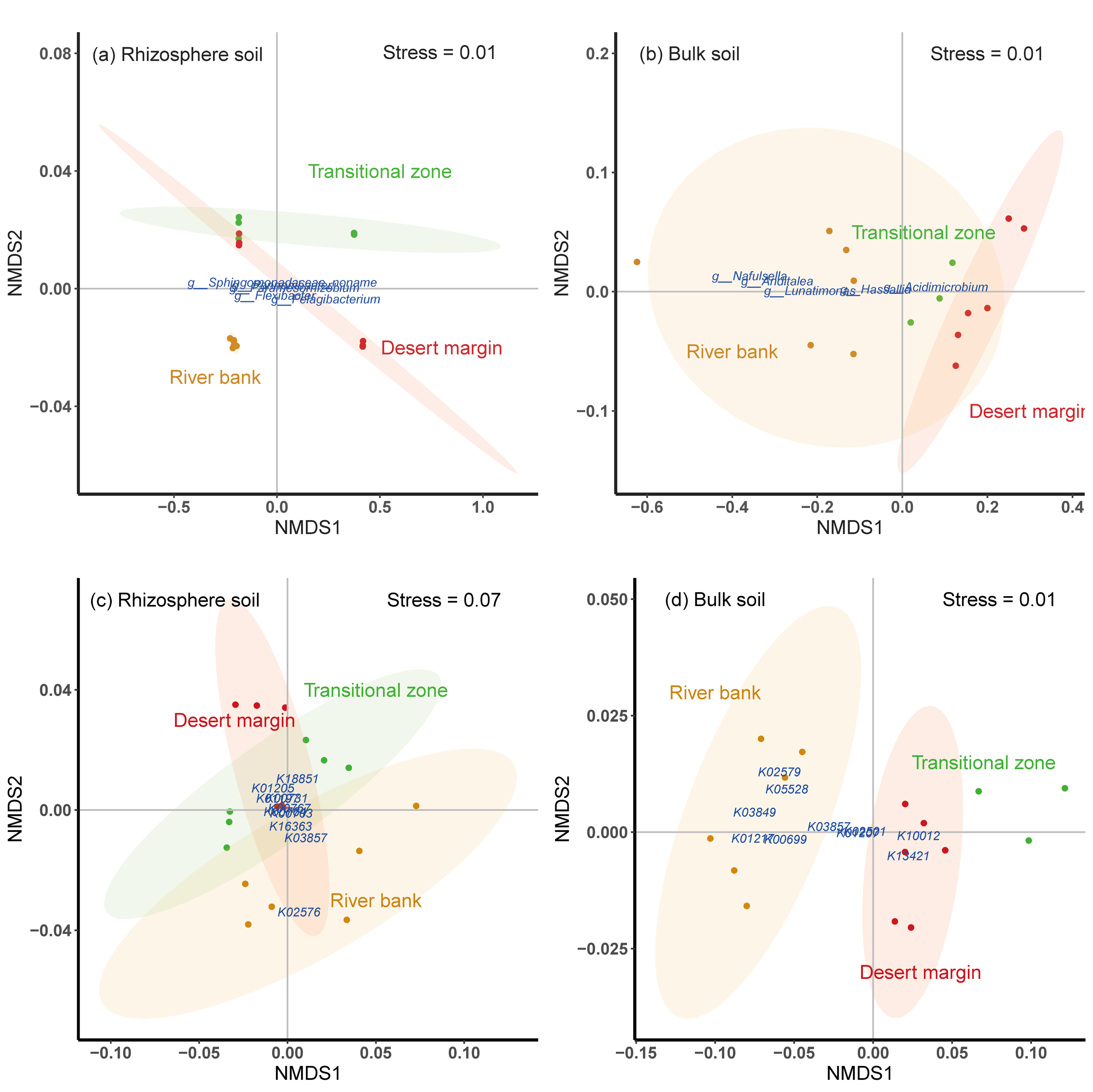
**

Supplementary Figure S3. Nonmetric multidimensional scaling (NMDS) analysis of microbial classification attributes (a, b) and functional attributes (c, d). Relevant information on the CAZy (carbohydrate active enzyme) database is available in Supplementary Table S3.
